# Supplementary material for: The DANish Disseminated Intravascular Coagulation (DANDIC) Cohort Study: Time Trends in Incidence and Short-Term Mortality
Source: J Clin Med. 2024 Oct 2;13(19):5896. doi: 10.3390/jcm13195896 (PMC11477667; doi:10.3390/jcm13195896)

Supplementary Materials

Supplementary Table S1. ICD-10 codes used to calculate Charlson Comorbidity Index scores.

Comorbidity	Points	ICD-10 codes
Acute myocardial infarction	1	I21, I22, I25.2
Congestive heart failure	1	I50
Peripheral vascular disease	1	I71, I73.9, I79.0, R02, Z95.8, Z95.9
Cerebral vascular accident	1	I60, I61, I62, I63, I64, I65, I66, G45.0, G45.1, G45.2, G45.8, G45.9, G46, G45.4, I67.0, I67.1, I67.2, I67.4, I67.5, I67.6, I67.7, I67.8, I67.9, I68.1, I68.2, I68.8, I69
Dementia	1	F00, F01, F02, F05.1
Pulmonary disease	1	J40, J41, J42, J43, J44, J45, J46, J47, J60, J61, J62, J63, J64, J65, J66, J67
Connective tissue disorder	1	M32, M34, M33.2, M05.3, M05.8, M05.9, M06.0, M06.3, M06.9, M05.0, M05.2, M05.1, M35.3
Peptic ulcer	1	K25, K26, K27, K28
Liver disease	1	K70.2, K70.3, K73, K71.7, K74.0, K74.2, K74.6, K74.3, K74.4, K74.5
Diabetes	1	E10.9, E11.9, E13.9, E14.9, E10.1, E11.1, E13.1, E14.1, E10.5, E11.5, E13.5, E14.5
Diabetes complications	2	E10.2, E11.2, E13.2, E14.2, E10.3, E11.3, E13.3, E14.3, E10.4, E11.4, E13.4, E14.4
Paraplegia	2	G81, G04.1, G82.0, G82.1, G82.2
Renal disease	2	N03, N05.2, N05.3, N05.4, N05.5, N05.6, N07.2, N07.3, N07.4, N01, N18, N19, N25
Cancer	2	C0, C1, C2, C3, C40, C41, C43, C45, C46, C47, C48, C49, C5, C6, C70, C71, C72, C73, C74, C75, C76, C80, C81, C82, C83, C84, C85, C88.3, C88.7, C88.9, C90.0, C90.1, C91, C92, C93, C94.0, C94.2, C94.3, C94.7, C95, C96
Metastatic cancer	3	C77, C78, C79, C80
Severe liver disease	3	K72.9, K76.6, K76.7, K72.1
AIDS/HIV	6	B20, B21, B22, B23, B24

Supplementary Table S2. ICD-10 codes used to assess the occurrence per year of the diseases associated with DIC in the entire Danish population aged 20 years and older.

	**ICD-10 codes**
Infection	A00-A49, A65-A99, B00–99, G00-G02, G04.2, G05-G07, I33, I38, I39.8, I40.0, I41.0–2, I52, J00-J22, J36, J39.0–2, J85-J86, K04.6–7, K35-K37, K61, K63.0, K65, K75.0, K81, K83.0, L00, N10, N30, N34.0, N39.0, N41.0–3, N45, N73,3, N75.1, N76.4
Malignancy	C00-C97, D46
Obstetric complications	O00, O03, O05, O06, O08, O11, O14, O15, O23, O36.4–5, O41.1, O42, O45, O60, O88.1
Cardiac arrest	I46
Trauma	S06.1, S06.5–6, S07, S08, S13.0, S17, S18, S23.0, S27.0–2 S28, S33.0, S33.4, S38, S47, S48, S53.2–3, S57, S58, S63.3–4, S67, S68, S77, S78, S87, S88, S97, S98, T04, T05, T09.6, T11.6, T13.6, T14.7,
Others	K85, T40, T63.0, T67.0, T78.0, T80.8–9

Supplementary Table S3. International Society on Thrombosis and Haemostasis and modified Japanese Association of Acute Medicine DIC scoring algorithms.

	**ISTH**		**JAAM**	
	**Value**	**Score**	**Value**	**Score**
**Platelet count (10^9/L)**<br>Reference range:<br>145-350 (men),<br>165-390 (women)	>100	0	≥120	0
	≥50 and ≤100	1	≥80 and <120 or >30% decrease within 24 hours	1
	<50	2	<80 or >50% decrease within 24 hours	3
**INR**<br>Refence range:<br><1.3	<1.3	0	>1.2	1
	≥1.3 and ≤1.6	1		
	>1.6	2		
**Fibrin D-dimer (mg/L)**<br>Reference range:<br>age-specific*	<1	0	<10	0
	≥1 and <10	2	≥10 and <20	1
	≥10	3	≥20	3
**Fibrinogen (µmol/L)**<br>Reference range:<br>5.5-12	≥2.94	0		
	<2.94	1		
**Antithrombin (IU/L)**<br>Reference range:<br>0.80-1.20x10^3			≥0.80	0
			<0.80	1

* 0-55 years: <0.50; 55-65 years: <0.60; 65-75 years: <0.70; 75-85 years: <0.80; 85-95 years: <0.90; 95-105 years: <1.00; 105-115 years: <1.10

Abbreviations: INR: International Normalized Ratio; ISTH: International Society on Thrombosis and Haemostasis; JAAM: Japanese Association of Acute Medicine

Supplementary Figure S1. Number of patients with a measurement of all DIC biomarkers on the same date grouped by positive and negative DIC scores.

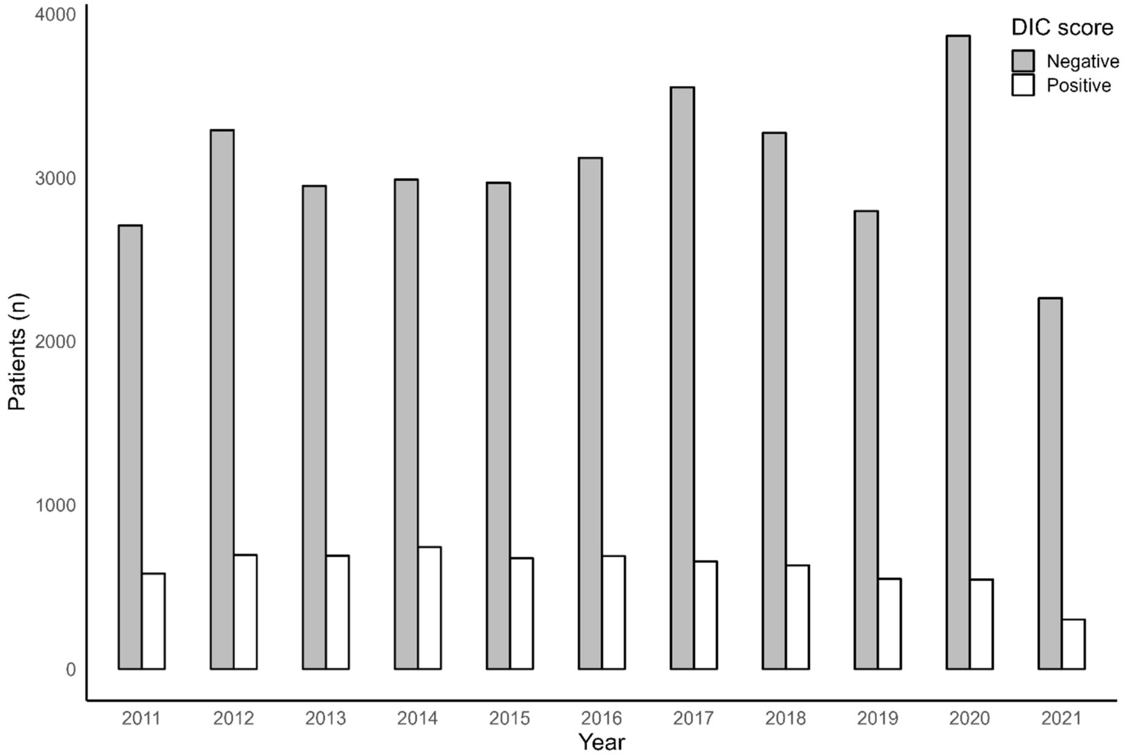

Supplementary Figure S2. Number of patients per year with a disease associated with DIC in Denmark from 2013 to 2020 among patients aged 20 years and older.

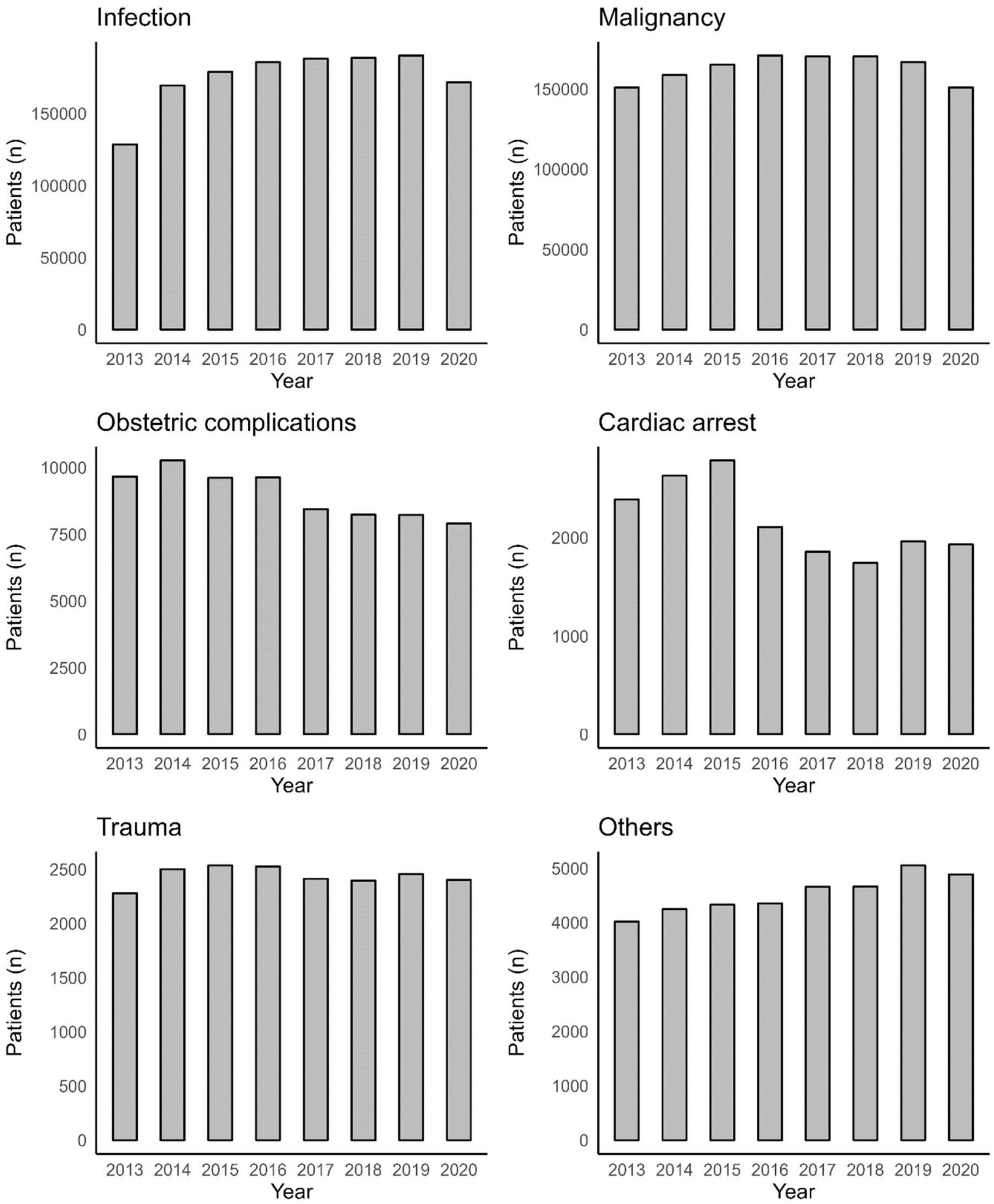

Supplement: Supplementary file 1 [file jcm-13-05896-s001.zip › jcm-3099268-supplementary.pdf]
